# Dietary Patterns and Their Association with Body Composition and Cardiometabolic Markers in Children and Adolescents: Genobox Cohort

**DOI:** 10.3390/nu12113424

**Published:** 2020-11-08

**Authors:** Miriam Latorre-Millán, Azahara I. Rupérez, Esther M. González-Gil, Alba Santaliestra-Pasías, Rocío Vázquez-Cobela, Mercedes Gil-Campos, Concepción M. Aguilera, Ángel Gil, Luis A. Moreno, Rosaura Leis, Gloria Bueno

**Affiliations:** 1 GENUD Research group, Instituto de Investigación Sanitaria de Aragón (IIS Aragón), Universidad de Zaragoza, 50013 Zaragoza, Spain; latorremiriam0@gmail.com (M.L.-M.); airuperez@unizar.es (A.I.R.); esthergg@ugr.es (E.M.G.-G.); albasant@unizar.es (A.S.-P.); lmoreno@unizar.es (L.A.M.); mgbuenol@unizar.es (G.B.); 2 Unidad de Endocrinología Pediátrica, Hospital Clínico Universitario Lozano Blesa, 50009 Zaragoza, Spain; 3 Instituto Agroalimentario de Aragón (IA2), 50013 Zaragoza, Spain; 4 Centro de Investigación Biomédica en Red de Fisiopatología de la Obesidad y Nutrición (CIBEROBN), Instituto de Salud Carlos III, 28029 Madrid, Spain; mercedes_gil_campos@yahoo.es (M.G.-C.); caguiler@ugr.es (C.M.A.); agil@ugr.es (Á.G.); 5 Departamento de Bioquímica y Biología Molecular II, Instituto de Nutrición y Tecnología de los Alimentos, Centro de Investigación Biomédica, Universidad de Granada, 18016 Granada, Spain; 6 Unidad de Gastroenterología, Hepatología y Nutrición Pediátrica, Grupo de Investigación Nutrición Pediátrica, Instituto de Investigación Sanitaria de Santiago de Compostela (IDIS), Complejo Hospitalario Universitario de Santiago, 15706 Santiago de Compostela, Spain; cobela.rocio@gmail.com; 7 Unidad de Metabolismo e Investigación Pediátrica, Hospital Universitario Reina Sofía, Instituto Maimónides de Investigación Biomédica de Córdoba (IMIBIC), 14071 Córdoba, Spain; 8 Instituto de Investigación Biosanitaria IBS.GRANADA, Complejo Hospitalario Universitario de Granada, 18014 Granada, Spain; 9 Unidad de Investigación en Nutrición, Crecimiento y Desarrollo Humano de Galicia (GALINUT), Universidad de Santiago de Compostela, 15706 Santiago de Compostela, Spain

**Keywords:** cluster analysis, obesity, diet, anthropometry, inflammation, oxidative stress, cardiovascular disease

## Abstract

Diet is a key factor for obesity development; however, limited data are available on dietary cluster analysis in children with obesity. We aimed to assess the associations between dietary patterns and obesity and several cardiometabolic markers. Anthropometry, bioelectrical impedance, blood pressure and plasma biomarkers of oxidative stress, inflammation and endothelial damage were determined in 674 Caucasian children, aged 5–16, with normal or excess weight. Using a food frequency questionnaire and cluster analysis, two consistent dietary patterns were shown, labeled as health conscious (HC) and sweet and processed (SP). The HC pattern included a greater proportion of participants with overweight/obesity than the SP cluster (80.1% vs. 63.8%). However, children with obesity within the HC cluster, showed less abdominal fat, through waist to hip (0.93 vs. 0.94) and waist to height (0.61 vs. 0.63) indexes (*p* < 0.01). Univariate general models showed several additional differences in cardiometabolic risk biomarkers in the global and stratified analyses, with a healthier profile being observed mainly in the HC cluster. However, multivariate models questioned these findings and pointed out the need for further studies in this field. Anyhow, our findings support the benefits of a healthy diet and highlight the importance of dietary patterns in the cardiometabolic risk assessment of children with overweight/obesity, beyond weight control.

## 1. Introduction

The World Health Organization (WHO) stated that the prevalence of overweight and obesity has risen dramatically worldwide from 1975 to 2016, as it has nearly triplicated among adults, and even more among children and adolescents (aged 5–19), increasing from 4% to 18% [1]. WHO also highlighted Spain among the European countries with the highest prevalence [1], and although national studies suggested that it has stabilized in the last few years, it continues to be high in this country, at over 40% (for children seven and eight years old) [2]. This is a concern as obesity is related to a wide range of negative health outcomes in adults and children [3]. In the pediatric age, priorities include considering diet in relation to excess weight and over-feeding complications [4]. High intake of energy-dense foods is a significant contributor to excess body mass index (BMI), being considered as a leading risk factor to the global burden of disease [5].

Dietary patterns analysis is considered one of the best dietary approaches [6,7], as it accounts for the overall diet, by including the interactive effect of individual food items, macro- and micro- nutrients, and bioactive compounds. It has been increasingly applied in recent years to evaluate diet and its relationship to health outcomes, linking specific dietary habits to chronic diseases, including obesity and related phenotypes such as body composition and cardiometabolic markers [6,7,8,9,10]. However, as some reviews have highlighted [11,12], the association between dietary patterns and BMI has shown inconsistent results in cross-sectional studies in children and adolescents. Additionally, there is still scarce literature on these populations regarding the associations between dietary patterns and other variables of body composition beyond BMI, and metabolic indicators, especially biochemical ones related to oxidative stress, inflammation or endothelial damage [11,12]. Indeed, in Spain the enKid [13], the ANIBES [14], the EsNuPi [15], and the SI! [16] studies had large sample sizes (n > 400), and all considered BMI exclusively as an obesity phenotype indicator in relation to dietary patterns (except SI! [16], which also included a few other parameters but in participants of a very small age range) and were not performed in a clinical care environment. Hence, there is little information available on dietary patterns related to obesity phenotype among children and adolescents.

Therefore, the main objective of this study was to assess the associations between dietary patterns identified by cluster analysis and obesity and several cardiometabolic markers of body composition, blood pressure, general metabolism, oxidative stress, inflammation and endothelial damage, in children and adolescents.

## 2. Materials and Methods

### 2.1. Study Sample

A total of 793 children from those recruited in the GENOBOX clinic cohort participated in this study after applying the inclusion and exclusion criteria. The GENOBOX study was carried out in three hospitals from cities located in different Spanish areas: Hospital Clínico Universitario de Santiago (Santiago de Compostela), Hospital Clínico Universitario Lozano Blesa (Zaragoza), and Hospital Universitario Reina Sofía (Córdoba). Participants were recruited after attending the hospital for diagnosis of minor disorders that were not confirmed after clinical and laboratory investigations or suspecting overweight or obesity. For the current study, children with any BMI classification were eligible, other inclusion criteria included: 5–16 years old, being Caucasian, absence of endogenous obesity, and having a minimal amount of useful dietary intake data. Exclusion criteria were disease and the use of medications that altered blood pressure, glucose or lipid metabolism, having exercised intensely in the 24 h previous to the examination, having participated in a research study in the last three months, having not signed the written informed consent, or not meeting the inclusion criteria.

All participants and their families were informed about the purpose of the study before giving their written consent. The study was developed following the Declaration of Helsinki recommendations (as well as the Edinburgh review) and was approved by the ethics committees of each participating center (Code IDs: Santiago 2011/198, Zaragoza 10/2010, Córdoba 01/2017).

### 2.2. Body Composition Indicators

Trained staff performed the body composition measurements, according to the International Society for the Advancement of the Kinanthropometry (ISAK) standardized procedures and criteria [17]. All measurements were made in the anatomical position and in underwear. Height, body circumferences and skinfolds were recorded at a 1 mm accuracy.

Height (floor to vertex distance) was measured with a standing stadiometer (SECA^®^ 225 model, Seca gmbh & co, Hamburg, Germany). Waist and hip circumferences were measured with a Cescorf^®^ inelastic tape (Cescorf Equipamentos para Esportes, Porto Alegre, Brazil). Waist to hip, and waist to height indexes were calculated. Skinfolds (biceps, triceps, subscapular and supraspinal) were determined with a caliper (Holtain^®^, Crosswell, UK). The sum of these four skinfolds was calculated to perform the subsequent analyses.

A scale adapted for children (BC420SMA, Tanita^®^, Tokio, Japan) was used to perform the bioelectrical impedance analysis (BIA) and to determine weight. Weight was recorded in kg with an accuracy of one decimal. BMI was calculated as the ratio between weight (kg) and the square of height (m^2^) and its *Z*-scores related to national reference values [18]. Children were classified as having normal weight, overweight or obesity, according to BMI by using the Cole et al. sex and age cut-offs for children [19]. BIA was used to obtain body fat mass (FM) and fat free mass (FFM) values (grams and percentage). Fat mass index (FMI) and free fat mass index (FFMI) *Z*-scores were calculated according to Wells et al. [20].

### 2.3. Blood Pressure

Systolic (SBP) and diastolic (DBP) blood pressure were measured twice in the participants’ right arm, using an electronic manometer (M6, HEM-7001-E, Omron^®^, Tokio, Japan), with a 5 min interval. If measures differed more than 20%, an additional measurement was taken. The mean value was calculated as the average of the two closest measurements.

### 2.4. Blood Samples and Biomarkers

Blood samples were collected from the antecubital vein after at least 8 h of overnight fasting. General biochemical analyses were done at the participating university hospitals using automatic analyzers. These analytes were: glucose (mg/dL), insulin (mU/L), triglycerides (TG, mg/dL), total cholesterol (mg/dL), high-density lipoprotein cholesterol (HDLc, mg/dL), low-density lipoprotein cholesterol (LDLc, mg/dL), creatinine (mg/dL), aspartate transaminase (AST, U/L), alanine transaminase (ALT, U/L), and gamma-glutamyl transferase (GGT, U/L).

Biomarkers of oxidative stress, inflammation and endothelial damage were determined in laboratories of the University of Granada. Tocopherols and carotenes levels (nmol/L) were analyzed by high-performance liquid chromatography [21], expressed as tocopherols/TG and carotenes/TG. Total plasma antioxidant capacity (TAC, mM Eq Trolox^®^) was studied using an antioxidant spectrophotometric assay kit (709001, Cayman Chemical^®^, Ann Arbor, MI, USA). Catalase activity (CAT, U/g hemoglobin) was determined in erythrocytes as described [22], by a colorimetric method (K033-H1, DetectX^®^ Arbor Assays, Ann Arbor MI, USA). The remaining biomarkers were analyzed with X-Map technology and LINCO-plexTM kits for human monoclonal antibodies (Linco Research, St Charles, MO, USA), using a Luminex^®^ 200TM device (Luminex Corporation, Austin, TX, USA), following the manufacturer’s instructions. The kits used were as follows: panel A of human serum adipokines (HADK1-61K-A-03): adiponectin (mg/L) and resistin (µg/L); panel B of human serum adipokines (HADK2-61K-B-07): leptin (µg/L), monocyte chemoattractant protein-1 (MCP-1, pg/L), and tumor necrosis factor alpha (TNFα, pg/L); panel 1 of human cardiovascular disease (CVD) (HCVD1-67AK-06): selectin (ng/L), soluble vascular cell adhesion molecule-1 (sVCAM-1, ng/L), myeloperoxidase (MPO, µg/L), and total plasminogen activator inhibitor-1 (tPAI-1, µg/L).

Additionally, two biochemical indexes were calculated: HDLc/LDLc, and the homeostasis model assessment for insulin resistant (HOMA-IR) [23].

### 2.5. Dietary Assessment

Dietary intake was assessed using a qualitative food frequency questionnaire (FFQ), which included common foods consumed in Spain. The children and their caregivers were interviewed by a trained dietician, and consumption frequency of each one of the 83 food items in the last four weeks was recorded as never or hardly ever; 1–3 times per month; once, 2–4 or 5–6 times per week; and once, 2–3, 4–6 or >6 times per day. These answers were converted into times per week (ranging 0 to 42) for their use in further analyses.

### 2.6. Covariates

Covariates were collected in a questionnaire that included questions regarding lifestyle behaviors, medical history, and socioeconomic status. Recruitment center, age and gender were recorded. Participants were classified as children or adolescents according to their age, since traditionally dietary guidelines establish a difference between school age and adolescence, and also energy requirements increase significantly from 12 years [24]. Maternal education reported by parents was classified according to the International Standard Classification of Education (ISCED) criteria, as low (primary school), medium (high school) or high (bachelor’s degree or higher) [25]. The regular performance of moderate-to-vigorous physical activity was evaluated from two questions: (1) “Does your child practice any extracurricular sport?” and (2) “Is your child member of any sports club?” [26]. If either one of the answers was positive, the child was considered a regularly active child, with the opposite, if both were negative, the child was considered a non-active child. In addition, pubertal stage was determined according to Tanner’s criteria [27] by a pediatrician. Children in stage I were considered as prepubertal, and children in stages II-V as pubertal.

### 2.7. Statistical Analyses

The sample size estimation was calculated for the GENOBOX study, based on the principal cardiometabolic risk factors associated with obesity, as previously described [28]. Figure S1 shows a flow diagram that reflects the evolution of the sample.

Dietary patterns were identified through cluster analysis (CA). First, a data cleaning process was performed. Initially, 793 children and adolescents with available FFQ data were included. The 83 food items were grouped into 44 study variables (as shown in Table S1). After removing individuals with more than 50% of missing values for those variables, 765 subjects remained for subsequent analyses. Multiple imputations were applied to estimate missing values using gender, age, BMI, and origin (recruitment center) as predictors for missing values, and the pooled data from the imputed databases were retrieved. Out of the 44 food items included in the FFQ, “meat substitute products and soy products” were excluded from the analysis as more than 95% of the subjects reported to consume them “never or almost never” or “1–3 times per month”. Correlations between the single items were calculated to assess multi-collinearity, and no redundant variables were identified. Standardized *Z*-scores were obtained for the remaining 43 food items, as variance differences of the variables may otherwise affect the resulting clusters. Additionally, univariate (*n* = 28) and multivariate (*n* = 63) outliers were removed. Finally, 674 subjects remained for the following analyses.

A combination of hierarchical and non-hierarchical CA was used to identify individuals with similar dietary patterns. First, hierarchical CA was performed using Ward’s method, based on squared Euclidian distances. Several possible cluster solutions were identified and compared to inform the next step, considering the coefficients and fusion level. A non-hierarchical k-means clustering procedure was used, specifying the number of clusters identified in the first step, using a random initial seed and ten iterations to further refine the preliminary solution by optimizing classification. The solution of the number of clusters was identified through the widely used dendrogram and elbow graphical methods. The dendrogram method illustrates the groupings derived from the application of the hierarchical clustering algorithm in an arboreal way, whereas the elbow method represents, in a linear way, the inertia (sum of the squared distances of each cluster object from its centroid) for the different solutions proposed, allowing a sudden change in the slope or flexion (elbow) coinciding with the appropriate number of solutions to be seen. The final cluster solution was selected based on interpretability, stability, and the proportion of the study population in each dietary pattern. Randomly splitting the database in two halves to repeat the same procedure in a subsample (50%) was used to examine the stability and reliability of the final solution, obtaining a Cohen’s kappa value of 0.950. Radar plots showing the maximum and minimum Z-score values of each dietary pattern were compared to describe the clusters and study their interpretability.

Additionally, further analyses were performed in the total sample, as well as stratifying by dietary pattern and subgroups according to BMI status (normal weight, overweight, obesity), age (children 5–11 years, adolescents 12–16 years), and gender (male, female). Normal distribution of the variables was assessed using the Kolmogorov–Smirnov test, and non-normally distributed variables (SBP, DBP, HDLc, resistin, TNFα, selectin, and sVCAM1) were transformed into a logarithm scale for analyses.

Means and standard deviation were calculated for the studied continuous variables, then Levene’s and Student’s t test were used for simple comparisons between pair of groups. Likewise, Pearson’s chi-square (χ^2^) test was used to study categorical variables.

Finally, differences in the means of body composition and cardiometabolic indicators by dietary pattern were estimated by models adjusted for gender, recruitment center, sport practice and Tanner stage. Additionally, maternal education and BMI Z-score were used as confounders in body composition and cardiometabolic variables, respectively. As some dependent variables might be related, to avoid the chance of making a type error I, we carried out a multivariate analysis of covariance (MANCOVA) for combined variables of the same group, taking into account the covariates. Levene’s test and Box’s M test were used for checking assumptions of homogeneity of variances and variance–covariance matrices, respectively. Later, as the second step for MANCOVA, we performed follow-up univariate analysis of covariance (ANCOVA) on stepwise generalized linear models (GLM) on each dependent variable and discriminant analysis.

All statistical analyses were carried out with SPSS 19.0, (IBM, Chicago, IL, USA). Graphics were performed with Excel 2016 (Microsoft, Redmon, Washington, DC, USA). 

## 3. Results

The general characteristics of the total study population and BMI ranged subgroups are shown in Table 1. Compared with the normal weight group, those with overweight/obesity were more likely to be female and to have mothers with a low or medium education level, and they showed significantly greater values for BMI and BMI Z-score. No differences by BMI status were found for pubertal stage and age.

### 3.1. Dietary Patterns

**Figure 1:**
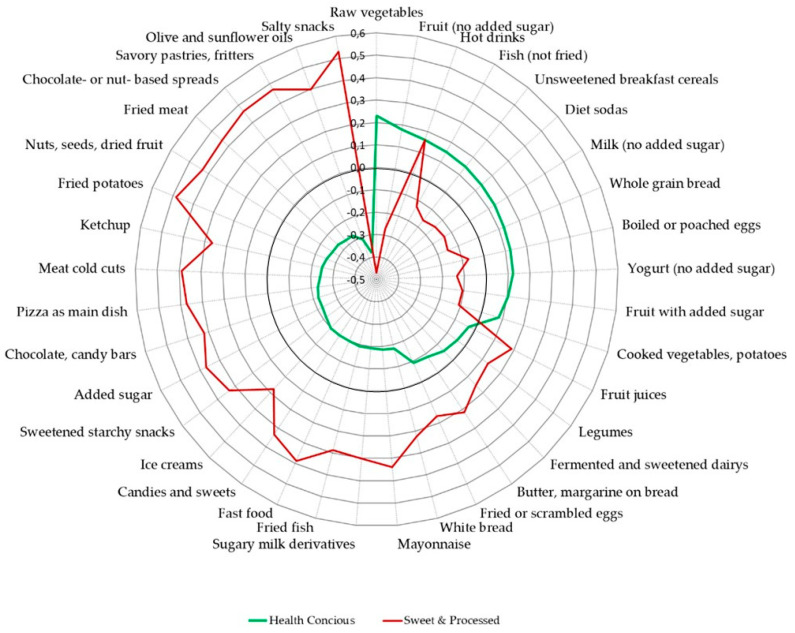
Radar plot of the significant differences (*p* < 0.05) in food frequency questionnaire (FFQ) items that characterize each dietary pattern in Spanish children and adolescents.

A two clusters solution for dietary patterns was considered the most interpretable and stable. These patterns were labeled as “Health Conscious” (HC) and “Sweet and Processed” (SP). Radar plots shown in Figure 1 and Table S2 show the differences in *Z*-scores of the food items for each pattern. The relative frequency of most food items differed significantly between the clusters. Compared with the SP pattern, the HC pattern showed significantly (*p* < 0.001) lower mean FFQ *Z*-scores for salty snacks, vegetable oils, savory pastries, chocolate or nut-based spreads, fried meat, nuts and seeds, fried potatoes, ketchup, cold cuts, pizza, chocolate, added sugar, sweetened starches, ice creams, candies and trinkets, fast food, fried fish, sweetened milk products, mayonnaise, white bread, butter and margarine. Whereas the foods showing the most significantly (*p* < 0.001) higher mean FFQ *Z*-scores in the HC pattern were raw vegetables, fruit with no added sugar, hot drinks, fish (not fried), unsweetened breakfast cereals, diet drinks and whole grain bread. There were no significant differences for sweetened breakfast cereals, water, rice and pasta, cheese, meat (not fried) and sweetened drinks.

**Figure 2:**
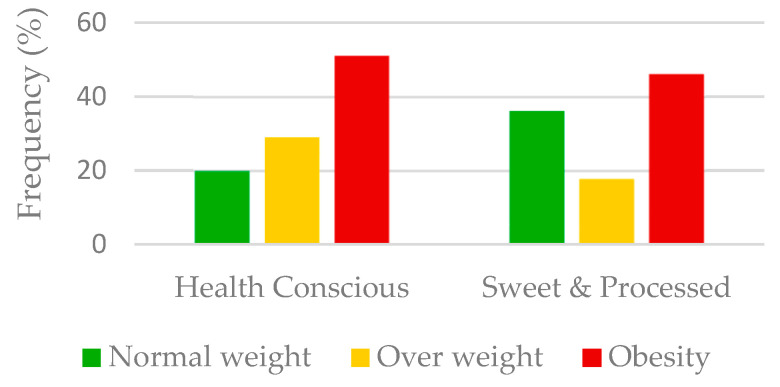
Frequency distribution of BMI subgroups in children and adolescents in each dietary cluster (GENOBOX cohort).

As the bar plot in Figure 2 shows, compared to the SP cluster, the HC cluster included a greater proportion of participants with overweight or obesity (29.0% and 51.1% vs. 17.7% and 46.1%, *p* < 0.001). In addition, Table 2 shows higher age, BMI and BMI Z-score in children allocated to the HC cluster than in those in the SP cluster, as well as more pubertals and adolescents. However, no significant differences were found between clusters related to gender and maternal education level. 

### 3.2. Obesity Related Cardiometabolic Risk Indicators and Dietary Patterns

When applying the multivariate analysis, there was a statistically significant difference between the dietary clusters on combined body composition variables (BMI Z-score, skinfold sum and hip circumference) after controlling for covariates (recruitment center, sport practice, pubertal stage, gender, and maternal education) in the total sample, F(3, 491) = 4,290, *p* = 0.005, Wilks’ λ = 0.974, partial η^2^ = 0.026 (Box’s M *p*-value = 0.062, Wilks’ λ *p*-value for discriminant analysis < 0.001). However, no significant results were found for the rest of the biomarkers.

Table 3 shows mean and standard deviation values for body composition parameters, blood pressure and circulating biomarkers for each cluster in the total sample and in subgroups by BMI status when GLM were applied. Significant differences were found in the total sample, compared to the SP cluster, being allocated to the HC cluster was associated with higher mean values for age, hip circumference, skinfold sum, carotenes/TG, catalase activity, leptin, and MPO; and lower mean values for DBP, resistin, TNFα, MCP-1, tPAI-1, and sVCAM-1.

When stratifying by BMI, additional differences between clusters were observed. In the normal weight subgroup, children in the HC cluster showed lower mean values for DBP and HDLc/LDLc index than those in the SP cluster. In the overweight subgroup, those allocated to the HC cluster showed higher mean values for age, BMI, BMI Z-score, weight, and hip circumference; and lower mean values for AST, GGT, catalase, tPAI-1 and selectin, than children in the SP cluster. Lastly, in the obesity subgroup, those allocated to the HC cluster showed lower mean values for waist to hip and waist to height indexes, and MCP-1, than children in the SP cluster.

Table S3 shows mean and standard deviation values for body composition parameters, blood pressure and circulating biomarkers for each dietary cluster, stratified by age and gender. On one hand, compared to the SP cluster, those younger children allocated to the HC cluster showed higher mean values for BMI, BMI Z-score, weight, hip and waist circumference, skinfold sum, FMI Z-score, ALT, GGT, carotenes/TG, leptin and adiponectin/leptin index; and lower mean values for catalase, MCP-1, tPAI-1, sVCAM-1 than those allocated to the SP cluster. Whereas adolescents allocated to the HC cluster showed higher mean values for waist to height index; and lower mean values for HDLc/LDLc index, adiponectin, TNFα and MCP-1 than those in the SP cluster. On the other hand, males allocated to the HC cluster showed higher mean values for hip circumference, skinfold sum, ALT, and leptin; and lower mean values for DBP, MCP-1 and tPAI-1 than those in the SP cluster. However, females allocated to the HC cluster showed higher mean values for age, waist to hip index, total cholesterol, LDLc, and MPO; and lower mean values for HDLc/LDLc index than those in the SP cluster.

## 4. Discussion

In the present study, we have observed that those children allocated to the HC pattern showed higher BMI than those in the SP pattern. However, being allocated to the HC cluster was associated with a mainly healthier cardiometabolic profile, although we will also discuss some conflictive results. Overall, these results suggest that other factors could influence health status beyond BMI and current food consumption frequency but also highlight that having a healthy dietary pattern could help prevent future cardiometabolic risk.

Dietary patterns have been previously explored using two types of analytical approaches. On the one hand, a priori driven methods, which focus on constructing dietary scores using a predefined combination of diet quality based on dietary guidelines. On the other hand, a posteriori exploratory methods, which reduce large datasets into smaller ones to summarize total dietary exposure by the use of multivariate statistical techniques. In relation to the latter group, CA has been increasingly applied in recent years, mainly because it provides an intuitive picture of the whole diet. CA reduces behaviors into patterns and categorizes individuals according to continuous and mutually exclusive differences. In contrast, principal component analysis (PCA) or reduced rank regression (RRR) assign scores of the derived factors to each individual according to the intercorrelations of behaviors, which can also be influenced by a larger subjective component in decision making, especially in the way in which the researchers code and define the dietary pattern groups [7]. Hence, CA allows the completion of global assessments of the quality of the diet referring to identifiable groups of individuals, as well as their association with health outcomes and nutritional biomarkers, in a more understandable and applicable way in the field of dietary recommendations [6].

The conducted CA showed two dietary patterns, HC and SP, in the present sample of Spanish children and adolescents, with opposite characteristics regarding the consumption of the main food and beverage groups. The HC pattern was characterized by having higher consumption frequency (CF) of fruits and vegetables, whole grains, and dairy, being closer to compliance with the FFQ dietary guidelines [29]. In contrast, the SP pattern was characterized by having higher CF of snacks, sweets, fat, and processed foods. According to previous reviews regarding the use of CA or other approaches to study children and adolescents’ dietary patterns [11,12], definitions and labels of unhealthy eating patterns are wide, although they have been mainly characterized by a high consumption of high energy dense foods and beverages or ready-to-eat food, similar to our SP pattern. Otherwise, healthy and HC dietary patterns have been quite frequently described too, with a consistent characterization similar to ours and are generally considered to be protective against weight gain [3]. Specifically, in Spain, there have been four previous studies including large samples of children or adolescents, which also found healthy dietary patterns in their study populations by using different approaches, labeling them as “healthy” (enKID [13,30] and SI! [16]), or “Mediterranean-like” (ANIBES [14,31,32] and EsNuPi [15]), including a high intake of vegetables, fruits and fish, similar to our HC. 

Regarding obesity, these healthy patterns in Spanish children and adolescents generated conflicting results. ANIBES [14,31,32] and enKID [13,30] found a negative association, EsNuPi [13] found different associations according to age, and SI! [15] found a positive association, as did we. Indeed, we found that the HC cluster included a higher proportion of children with obesity (51.1% vs. 46.1%) and overweight (29.0% vs. 19.7%), and a lower proportion with normal weight (19.9% vs. 36.2%) than the SP cluster. However, there are also abroad studies that observed this counterintuitive cluster’s association in children and adolescents, such as a healthy one associated with a higher percentage of overweight/obesity in a cross-sectional study in Europe [33]. Similar findings have been reported through both cross-sectional and longitudinal approaches, in which healthier patterns were found to be related to higher BMI and BMI gain in Norway [34] and to higher obesity odds ratios in the U.S. [35]. In addition, it is interesting to note that children with excess weight were included in a high proportion in both clusters. This can be explained by the higher proportion of children with excess weight in the total sample. However, a second potential conclusion can be drawn, in which there could be two types of children with obesity, those who are conscious of the importance of a healthy diet and those who consume a lot of processed foods and sweets. The association between healthy dietary patterns and obesity has already been discussed in previous reviews [11,12], where all kinds of directions have been described, as well as highlighting the difficulty in comparing studies. In fact, explaining this controversy would require accounting for all determinants of energy balance and their interactions, as well as for differences in methodology, including the specific studied sample and bias [6,11,12].

First, associations such as the one described in the present study have been previously explained by other authors as reverse causation. This has been attributed to an attempt by the individuals to control their excess weight or the disease burden, and patterns have been described and labeled related to restrictive eating behaviors [11,12]. Indeed, HC patterns are in agreement with several interventions for obesity treatment, which recommend a high intake of foods considered to be protective against weight gain, such as vegetables and fruits, and a low intake of foods considered obesogenic, with a high fat and carbohydrate content [4,12,36,37].

Interestingly, sampling differences with previous studies are not negligible, as most of these were not based on cohort studies conducted in a clinical setting, but population cross-sectional studies (frequently using random multistage census- and school-based sampling procedures). In contrast, the present study has been performed in a specialized hospital environment and included subjects with excess weight attending health care services. This could imply a higher interest of the participants in improving their health, since non-attendance to health care visits has been associated with long-term excess weight in school children [38].

However, it could be possible that subjects with excess weight allocated to the HC cluster had some negative dietary-related behaviors not considered in the current analysis, which could contribute to a high energy imbalance. Indeed, a review highlighted the co-occurrence of both healthy and unhealthy behaviors, or even compensatory ones, or the use of ineffective dieting methods [35]. These behaviors could include differences in portion size, preparation, serving style, skipping breakfast or other meals, and having a high total energy intake. In fact, children with excess weight tend to have higher energy intake requirements, due in part also to higher fat-free mass [35]. However, in our study, we found no difference in FFMI between children from both clusters.

Otherwise, our analysis accounted for the main non-dietary confounders, considering physical activity due to its influence on obesity, along with diet [1], and the high prevalence of insufficient physical activity in Spanish adolescents (around three quarters) [39]. We also included origin (recruitment center) and maternal education level as covariates, as the main socio-economic and demographic factors in obesity susceptibility [30,40]. We found maternal education level was associated with obesity, but not to the HC cluster, as observed in previous studies [13,41]. Finally we took into account gender, age and puberty stage, due to the existing differences in obesity prevalence (higher in 6–13-year-old boys) [1,30], physiology of eating [42,43], cognitive behaviors [44,45], and the effect of diet on development [46]. Indeed, we found a HC cluster with a higher proportion of females and younger children, in agreement with previous literature [11], and with a lower consumption frequency of food puberty accelerators, such as sweetened soft beverages and meat products [46]. Somehow, as could be expected, we observed small differences in the results when stratifying the analyses between clusters by age or puberty, suggesting a possible presence of this effect on our findings.

Moreover, some authors consider the report bias to explain this association between obesity and a healthy pattern. They could include socially acceptable responses tending to under- or over- report food intake, or omit some foods they perceived as unhealthy, which are more common among subjects with an unhealthy diet, excess weight and males [47,48]. Additionally, reports about meals out of parental control would be missed as information was provided mainly by parents, while both a more frequent use of the school meals canteen (associated with healthier dietary intakes [49]) and a low family presence during the main meals have been reported in Spanish children and adolescents [50,51], with food intakes outside of parental control increasing with age [52]. However, our study used trained dieticians/nutritionists and imputation to minimize missing data, and, more importantly, we also found associations between the identified dietary patterns and several fat distribution and cardiometabolic markers reflecting a better health profile in all the subgroups of the HC cluster (including BMI as a covariate in the biomarkers analysis), so a strong effect of these bias reports should not be considered in the present study.

When analyzing the wide range of cardiometabolic risk biomarkers, we must take into consideration that some of the observed differences between the clusters may be due to chance. With this in mind, we conducted a multivariate analysis. Its results allowed us to support the differences related to body composition, but not those associated with the rest of biomarkers. However, the use of several dichotomous or ordinal variables as covariates (recruitment center, sport practice, gender, and maternal education) could have subtracted strength from the model. Thus, further studies are encouraged to search for the potential association of diet with these cardiometabolic risk biomarkers using more suitable continuous covariates.

Despite the lack of overall association, the individual generalized linear models showed interesting findings. Compared to the SP cluster, the HC cluster showed higher several subcutaneous and general fat mass indicators beyond BMI especially in those with overweight, children and males when stratifying analyses by BMI, age, and gender, respectively. However, after stratifying by BMI, age and gender, the SP cluster was found to be associated with a worse cardiometabolic profile. Within those in the obesity subgroup, participants allocated to the SP cluster showed higher indicators of abdominal/visceral fat mass (waist to hip and waist to height indexes), and therefore, a worse phenotype related to higher metabolic risk [53,54,55] than those in the HC cluster. Although it is the first time that this scenario has been described in Spanish children and adolescents with excess weight, our findings are supported by the available literature. Previous studies have reported a stronger influence of diet on fat mass than on muscle mass in overweight children [9], which is in line with our findings, since we found no differences related to fat free mass between the identified dietary clusters. However, other authors have described the positive association between BMI and healthy dietary patterns as being mediated by a higher muscle mass [56]. In contrast, when stratifying the body composition analysis by age and gender, we found some confusing results, as abdominal fat indicators such as waist to hip and waist to height were higher in the HC clustered females and adolescents, respectively. As other authors had found healthy and restrictive patterns to be more likely in this population [11,12], and considering the lower levels of physical activity in Spanish female adolescents [39], this could be explained in terms of the same reverse causation. This way, it could be possible that higher abdominal fat makes this population subgroup more conscious to follow a healthy dietary pattern. In addition, we also find the HC cluster associated with a worse lipid profile specifically in these subgroups, allowing us to suggest that the dietary pattern may less strongly influence a worse cholesterol metabolic profile than type of fat distribution (and physical activity levels) [57]. The SP cluster showed a worse CVD profile, indicated through higher DBP figures, when analyzed in the total population and the normal weight group, in agreement with a previous analysis carried out by our team, which identified a higher DBP in children with a higher consumption frequency of energy dense salty foods [58]. Additionally, the SP cluster was associated with a mainly worse metabolic phenotype through several biochemical indicators, both in the total sample and the age- gender- and BMI-stratified analyses. Hence, although the HC cluster was associated with higher obesity subcutaneous indicators (especially when analyzing the total sample and the overweight subgroup), it was also associated with a better phenotype in terms of oxidative stress, inflammatory and endothelial damage biomarkers. For example, compared to those with a SP pattern in the total sample, children with a HC pattern showed higher serum levels of carotenes/TG, as well as lower resistin, TNFα, MCP-1, tPAI-1, sVCAM1, although higher leptin and MPO. Moreover, the difference in MCP-1 was also found in the subgroup of participants with obesity, as well as in children, adolescents, and males, in the stratified analyses.

Regarding these results, the HC pattern was characterized by high frequency of consumption of vegetables, fruit, fish, low-fat dairy products, with a high content in antioxidants, micronutrients, fiber, and essential fatty acids. These exert antioxidant, anti-inflammatory and antithrombotic effects [59] and are known to contribute to the delay of CVD initiation and progression [59]. In this way, our results are in agreement with the previous ones of our team [60], showing plasma levels of carotenes/TAG to be correlated with the consumption frequency of carotene-rich foods in normal weight children, and decreased by a cumulative effect of obesity and unhealthy metabolic status, as well as negatively associated with BMI status, endothelial and glucose metabolism biomarkers in children and adolescents. In contrast, the SP pattern was characterized by high frequency of consumption of food items with a higher content in saturated fatty acids, salt, high glycemic index and low content in fiber and micronutrients. This kind of food contributes to higher levels of circulating free fat acids and glucose, and consequently to the increased generation of free radicals, which deplete the antioxidant defense systems and further contribute to inflammation, causing disruption in several metabolic pathways. The SP pattern is similar to other ones often labeled as western patterns, which have been related to undesirable health outcomes, including central obesity and weight gain, with moderate evidence in children and adolescents, but strong evidence in adults [12]. Indeed, although with a shorter list of biomarkers, a previous review on dietary patterns studies worldwide [12], and other European prospective studies using CA [10,11,12,13,14,15,16,17,18,19,20,21,22,23,24,25,26,27,28,29,30,31,32,33,34,35,36,37,38,39,40,41,42,43,44,45,46,47,48,49,50,51,52,53,54,55,56,57,58,59,60,61] have described similar associations in children and adolescents.

Our phenotype related findings support an effect of diet on the molecular mechanisms related to the “obesity paradox”, in which adipose tissue expansion is feasible without the accompanying adipocyte dysfunction [62], in children and adolescents. Indeed, mechanisms involving oxidative stress and inflammation have been described in childhood obesity [63], as well as the modulating role of diet over them [59,64,65,66]. Thus, our findings suggest that a HC dietary pattern can be associated with the metabolically healthy phenotype and a low cardiometabolic risk in children and adolescents with excess weight through both classic and novel indicators—most of them not commonly available in routine health control procedures. Otherwise, we found a SP pattern present in a high proportion of children and adolescents associated with a worse phenotype, both through fat distribution and cardiometabolic markers.

Limitations of this study must be acknowledged. The cross-sectional design does not allow the establishment of causal associations. Selection bias also cannot be ruled out given the voluntary nature of participation, which may involve under representation of certain population groups such as those who have no need for care from the social services [38] (possibly due to not being aware of having a health problem or, even if they were aware, this could make them want to avoid the negative results from an evaluation because they do not want the confirmation or to hear it from others, or to accept the consequences or initiate a change or intervention), or those with lower parental education levels or higher incomes. The inclusion of only Caucasian children could also be seen as a limitation, as dietary patterns may differ according to race and ethnicity, so this work does not reflect all Spanish children and adolescents. However, since the Caucasian ethnic group is the one that predominates our environment, it was considered as an inclusion criterion to homogenize the study population. The direction of these possible selection biases cannot be predicted as no information on non-participants is available. The possible effect of some reporting bias has already been discussed above. The used FFQ was not quantitative so it did not assess the total food intake, although when comparing dietary patterns obtained with and without energy adjustment it is unlikely that energy intake influences the membership results, as found by other authors [6,7]. In addition, it only covered the previous four weeks, and potential differences due to seasonality could have affected the results. However, reports and measurements were performed in the same period of time. Body composition measurement by BIA is not as accurate as other methods (such as DXA or BodPod®), but it is easier to apply when studying large clinical populations. Physical activity was assessed according to two questions characterizing sports activity, which could miss children that devote a substantial amount of time to recreational physical activity outside of planned sport lessons. Finally, the non-significant multivariate analysis regarding cardiometabolic risk biomarkers forces the individual significant findings to be considered as preliminary observations, which could be due to chance, and therefore that should be investigated further in future studies.

As for strengths, to our knowledge, this is the first Spanish study applying cluster analysis to identify dietary patterns in a cohort study on childhood obesity. Likewise, the use of a wide range of body composition and biochemical cardiometabolic risk indicators should also be considered a strength as there is a lack of studies assessing the effect of diet on these cardiometabolic risk indicators. In addition, the study was performed using standardized and harmonized information from clinical care centers of three different Spanish regions, providing comparable food consumption frequency estimates in a large sample size.

## 5. Conclusions

Our results suggest a mainly better cardiometabolic health for those children and adolescents who follow a healthy dietary pattern independently of BMI, and especially for those male children with overweight (who are the subpopulation with the highest prevalence of excess weight); however, multivariate analyses call these findings into question. Likewise, there is an HC dietary pattern that could be associated with a worse fat distribution and cholesterol metabolism in females and adolescents, which requires us to take into account further considerations and involved factors. Otherwise, there is also a SP dietary pattern linked to a mainly worse cardiometabolic profile that could lead to greater excess weight and worse health status in overweight children and adolescents. Consequently, our findings highlight the importance of the association between dietary patterns and cardiometabolic risk markers related to oxidative stress, inflammation, and endothelial damage, which may be already present in childhood, and determine the appearance of future cardiometabolic diseases. Longitudinal studies would be useful to corroborate and evaluate long-term implications of these findings. Diet along with other approaches should be considered in prevention studies aiming to reduce cardiometabolic risk, beyond weight control.

## Figures and Tables

**Table 1:** Descriptive characteristics of children and adolescents by body mass index (BMI) status (GENOBOX cohort).

	All *n* = 674 (100%)	Normal Weight *n* = 178 (26.4%)	Overweight and Obesity *n* = 496 (73.6%)	*p*
Gender				**0.018**
Male	307 (45.5%)	95 (53.4%)	212 (42.7%)	
Female	367 (54.5%)	83 (46.6%)	284 (57.3%)	
Pubertal stage (Tanner)				0.163
Prepubertal	333 (49.4%)	96 (53.9%)	237 (47.8%)	
Pubertal	341 (50.6%)	82 (46.1%)	259 (52.2%)	
Maternal education level				**<0.001**
Low	58 (8.9%)	9 (5.2%)	49 (10.3%)	
Medium	480 (73.8%)	116 (66.7%)	364 (76.5%)	
High	112 (17.2%)	49 (28.2%)	63 (13.2%)	
Age				0.273
Children (5–11 years)	439 (65.1%)	122 (68.5%)	317 (63.9%)	
Adolescents (12–16 years)	235 (304.9%)	56 (31.5%)	179 (36.1%)	
Age (years) Mean ± SD	10.7 (2.5)	10.5 (2.7)	10.7 (2.5)	0.297
BMI				**<0.001**
Normal weight	178 (26.4%)	178 (100%)	0 (0%)	
Overweight	165 (24.5%)	0 (0%)	165 (33.3%)	
Obesity	331 (49.1%)	0 (0%)	331 (66.7%)	
BMI (kg/m^2^) Mean (SD)	24.0 (5.6)	17.3 (2.3)	26.4 (4.3)	**<0.001**
BMI *Z*-score (kg/m^2^) Mean (SD)	1.8 (1.7)	−0.3 (0.6)	2.6 (1.3)	**<0.001**

*p*: significance of the χ^2^ test for categorical variables and Student’s *t* test for continuous variables, assessing differences between normal weight and overweight/obese groups. Bold letters in *p* values mean significant differences between BMI subgroups. Abbreviations: BMI, body mass index; SD, standard deviation.

**Table 2:** Descriptive characteristics of Spanish children and adolescents by dietary cluster (GENOBOX cohort).

	Health Conscious *n* = 403 (59.8%)	Sweet and Processed *n* = 271 (40.2%)	*p*
Gender			0.102
Male	175 (43.4%)	132 (48.7%)	
Female	228 (56.6%)	139 (51.3%)	
Pubertal stage (Tanner)			**0.019**
Prepubertal	184 (45.7%)	149 (55.0%)	
Pubertal	219 (54.3%)	122 (45.0%)	
Maternal education level			0.288
Low	29 (7.5%)	29 (11.1%)	
Medium	291 (75.0%)	189 (72.1%)	
High	68 (17.5%)	44 (16.8%)	
Age			**0.004**
Children (5–11 years)	245 (60.8%)	194 (71.6%)	
Adolescents (12–16 years)	158 (39.2%)	77 (28.4%)	
Age (years) Mean (SD)	10.9 (2.5)	10.3 (2.5)	0.002
BMI			**<0.001**
Normal weight	80 (19.9%)	98 (36.2%)	
Overweight	117 (29.0%)	48 (17.7%)	
Obesity	206 (51.1%)	125 (46.1%)	

*p*: significance of the χ^2^ test for categorical variables and Student’s t test for continuous variables, assessing differences between dietary clusters. Bold letters in *p* values mean significant differences between dietary clusters. Abbreviations: BMI, body mass index; SD, standard deviation.

**Table 3:** Mean differences of cardiometabolic and health risk indicators between dietary clusters in the three BMI subgroups of children and adolescents (GENOBOX cohort).

	All (*n* = 674)		Normal Weight (*n* = 178)		Overweight (*n* = 165)		Obesity (*n* = 331)	
	Health Conscious	Sweet and Processed	Health Conscious	Sweet and Processed	Health Conscious	Sweet and Processed	Health Conscious	Sweet and Processed
	(*n* = 403)	(*n* = 271)	(*n* = 80)	(*n* = 98)	(*n* = 117)	(*n* = 48)	(*n* = 206)	(*n* = 125)
Age (years)	**10.9 (2.5)**	10.3 (2.5) **	10.8 (2.9)	10.2 (2.5)	**11.7 (2.1)**	10.2 (2.1) ***	10.5 (2.5)	10.4 (2.6)
Body composition indicators								
BMI (kg/m^2^)	24.6 (5.1)	23.2 (6.3)	17.7 (2.6)	17.0 (2.0)	**23.7 (2.4)**	21.8 (2.3) **	27.7 (3.9)	28.5 (4.4)
BMI *Z*-score (kg/m^2^)	2.0 (1.6)	1.6 (1.9)	−0.21 (0.56)	−0.33 (0.54)	**1.44 (0.49)**	1.22 (0.42) *	3.11 (1.06)	3.35 (1.18)
Body mass (kg)	54.6 (18.5)	49.5 (20.6	38.7 (14.0)	34.3 (11.0)	**54.4 (12.2)**	45.9 (11.9) *	60.9 (19.2)	62.8 (20.3)
Hip circumference (cm)	**90.2 (13.6)**	83.2 (15.2) **	76.5 (11.4)	72.0 (9.5)	**91.2 (9.0)**	83.5 (8.6) **	95.5 (12.8)	95.3 (13.7)
Waist circumference (cm)	82.3 (15.0)	77.1 (17.6)	64.8 (11.8)	60.8 (5.9)	81.7 (9.7)	75.4 (9.4)	89.5 (12.6)	91.3 (14.4)
Waist to hip index	0.56 (0.08)	0.54 (0.10)	0.85 (0.08)	0.85 (0.07)	0.90 (0.08)	0.90 (0.07)	**0.93 (0.07)**	0.94 (0.09) **
Waist to height index	83.7 (30.1)	68.1 (35.7)	0.45 (0.05)	0.44 (0.04)	0.54 (0.05)	0.52 (0.04)	**0.61 (0.06)**	0.63 (0.06) **
Skinfold sum (mm)	**38.3 (10.0)**	33.9 (9.5) **	42.1 (18.7)	37.2 (17.3)	88.4 (17.7)	77.0 (22.1)	102.4 (18.8)	103.0 (22.6)
FMI *Z*-score (kg/m^2^)	11.6 (5.1)	10.4 (5.6)	3.5 (2.7)	2.7 (1.8)	6.4 (2.3)	5.7 (2.4)	9.7 (4.06)	9.3 (4.0)
FFMI *Z*-score (kg/m^2^)	10.9 (2.5)	10.3 (2.5)	8.1(4.2)	7.2 (3.9)	11.6 (4.7)	10.3 (4.1)	13.6 (4.9)	14.7(5.2)
Cardiometabolic indicators								
Blood pressure								
SBP (mm Hg) ^	109 (13)	108 (14)	104 (12)	100 (12)	108 (12)	106 (13)	112 (14)	121
DBP (mm Hg) ^	**65 (11)**	66 (10) *	**61 (9)**	63 (10) *	64 (11)	63 (8)	67 (11)	121
General metabolic biomarkers								
Glucose (mg/dL)	84 (8)	86 (8)	84 (8)	86 (7)	85 (8)	88 (8)	84 (8)	85 (8)
Insulin (mU/L)	12.20 (8.42)	11.52 (9.92)	8.00 (4.54)	7.27 (4.87)	11.11 (7.78)	10.55 (6.12)	14.47 (9.18)	15.4 (12.44)
HOMA-IR	2.57 (1.84)	2.48 (2.18)	1.68 (1.00)	1.56 (1.08)	2.35 (1.73)	2.36 (1.53)	3.05 (2.00)	3.27 (2.70)
TG (mg/dL)	69 (34)	69(35)	57(23)	54 (23)	66 (30)	77 (44)	76(38)	77 (34)
Cholesterol (mg/dL)	165 (30)	161 (28)	169 (26)	164 (28)	166 (33)	163 (31)	162 (30)	159 (26)
LDLc (mg/dL)	97 (26)	92 (25)	95 (22)	87 (26)	99 (30)	93 (25)	97 (26)	94 (24)
HDLc (mg/dL) ^	50 (13)	55 (15)	59 (13)	65 (15)	49 (11)	54 (14)	47 (12)	47 (11)
HDLc/LDLc index	0.61 (0.46)	0.81 (0.67)	**0.66 (0.22)**	0.94 (0.6) **	0.59 (0.43)	0.78 (0.65)	0.6 (0.54)	0.72 (0.73)
AST (U/L)	22 (9)	23 (7)	24 (8)	25 (6)	**20 (6)**	24 (9) *	22 (10)	22 (7)
ALT (U/L)	20 (12)	19 (11)	17 (9)	16 (6)	18 (9)	21 (21)	22 (14)	20 (8)
GGT (U/L)	12 (7)	13 (7)	10 (3)	10 (3)	**11 (5)**	14 (13) *	14 (9)	15 (5)
Oxidative stress biomarkers								
Carotenes/TG	**1.71 (1.65)**	1.53 (1.25) *	2.70 (2.32)	2.55 (1.56)	1.43 (1.12)	1.34 (0.81)	1.38 (1.36)	1.00 (0.76)
Tocopherols/TG	0.14 (0.07)	0.15 (0.07)	0.17 (0.07)	0.19 (0.07)	0.13 (0.06)	0.12 (0.06)	0.13 (0.08)	0.13 (0.05)
TAC (mM Eq Trolox^®^)	2.05 (0.87)	2.09 (0.91)	1.88 (0.66)	1.99 (0.75)	2.06 (0.85)	2.19 (1.13)	2.11 (0.97)	2.17 (0.96)
Catalase (U/g Hb)	**164.68 (103.66)**	163.94 (153.67) **	119.65 (71.23)	136.22 (119.74)	**165.71 (83.01)**	232.91 (283.63) *	180.90 (118.02)	162.27 (74.02)
Adipokines and biomarkers of inflammation and endothelial damage								
Adiponectin (mg/L)	14.58 (8.67)	15.18 (8.31)	17.27 (11.37)	17.08 (9.52)	14.91 (7.86)	14.51 (8.86)	13.13 (7.31)	13.51 (6.05)
Leptin (ug/L)	**15.56 (12.96)**	14.09 (15.82) **	4.59 (4.89)	3.95 (4.26)	12.44 (5.83)	12.47 (9.57)	22.47 (14.32)	25.13 (18.36)
Resistin (ug/L) ^	**20.18 (14.89)**	21.06 (14.47) *	24.07 (21.24)	23.85 (17.18)	18.43 (10.37)	18.80 (14.73)	19.4 (13.27)	19.24 (10.32)
TNFα (ng/L) ^	**2.82 (1.72)**	2.89 (1.58) *	2.27 (1.20)	2.4 (1.34)	2.67 (2.11)	3.26 (1.95)	3.15 (1.59)	3.23 (1.48)
MCP-1 (ng/L)	**88.47 (37.69)**	92.81 (38.84) **	83.54 (28.28)	85.21 (39.62)	88.01 (48.11)	89.70 (32.51)	**91.01 (34.32)**	102.26 (39.22) *
tPAI-1 (ug/L)	**22.9 (14.29)**	25.48 (17.31) ***	16.31 (12.21)	17.8 (12.94)	**21.76 (12.64)**	28.85 (17.23) **	26.61 (14.93)	31.92 (18.34)
Selectin (ug/L) ^	27.34 (14.7)	30.38 (16.59)	27.86 (15.66)	24.51 (11.69)	**22.45 (11.9)**	34.89 (20.32) *	31.06 (15.25)	33.6 (17.38)
sVCAM-1 (mg/L) ^	**1.04 (0.32)**	1.14 (0.25) *	1.07 (0.33)	1.20 (0.27)	1.00 (0.27)	1.15 (0.28)	1.04 (0.34)	1.07 (0.22)
MPO (ug/L)	**39.99 (83.94)**	32.95 (42.29) *	42.25 (77.48)	36.98 (50.22)	27.96 (29.79)	29.97 (39.36)	46.15 (105.63)	30.18 (33.95)

^ Logarithm transformed variable used for analyses. Bold letters mean significant differences (*: *p* ≤ 0.05; **: *p* ≤ 0.01; ***: *p* ≤ 0.001) between dietary patterns in the stepwise generalized linear models adjusted for recruitment center, sport practice, pubertal stage, gender and, additionally, maternal education level for body composition or BMI Z-score for metabolic variables. Student’s t-test was used to analyze age differences. Abbreviations: BMI: body mass index; circ.: circumference; FMI: fat mass index; FFMI, free fat mass index: SBP: systolic blood pressure; DBP: diastolic blood pressure; HOMA-IR: homeostatic model assessment for insulin resistance; TG: triglycerides, HDLc: high-density lipoprotein cholesterol; LDLc: low-density lipoprotein cholesterol; AST: aspartate transaminase; ALT: alanine transaminase; GGT: gamma-glutamyl transferase; TAC: total antioxidant capacity; TNFα: tumor necrosis factor alpha; MCP-1: monocyte chemoattractant protein-1; tPAI-1: total plasminogen activator inhibitor-1; sVCAM1: soluble vascular cell adhesion molecule-1; MPO: myeloperoxidase.

## Supplementary Materials

The following are available online at https://www.mdpi.com/2072-6643/12/11/3424/s1, Figure S1: Flow diagram of the sample, Table S1: Individual food and beverages included in each of the food items used for the cluster analysis in Spanish children and adolescent (GENOBOX study), Table S2: Relative food and beverages frequencies of consumption by dietary cluster in Spanish children and adolescents (GENOBOX study), Table S3: Main differences related to cardiometabolic risk indicators and health between dietary clusters in age and gender subgroups of children and adolescents (GENOBOX study).
