# Supplementary material for: Dietary Patterns and Their Association with Body Composition and Cardiometabolic Markers in Children and Adolescents: Genobox Cohort
Source: Nutrients. 2020 Nov 8;12(11):3424. doi: 10.3390/nu12113424 (PMC7695147; doi:10.3390/nu12113424)
Supplement: Supplementary file 1 [file nutrients-12-03424-s001.pdf]

Figure **S1.** Flow diagram of the sample

793 children and adolescents with FFQ data (GENOBOX cohort)

→ 28 participants removed because of >50% of missing values for FFQ variables

765

→ 91 subjects removed due to FFQ outliers

- 28 univariate outliers (>5DE relative food frequencies of consumption)
- 63 multivariate outliers (unsatisfactory Mahalanobis test)

674 participants finally included in the analysis

Table S1. Individual food and beverages included in each of the food items used for the cluster analysis in Spanish children and adolescent (GENOBOX study).

**Food item**	**Foods included**
Raw vegetables	Raw vegetables, usually mixed in a salad (e.g. cucumber, lettuce, tomato).
Fruit with no added sugar	Fresh fruit (also freshly squeezed, fruit smoothie) with no added sugar.
Hot drinks	Coffee, tea, infusions
Fish (not fried)	Fresh or frozen fish, (not fried)
Unsweetened breakfast cereals	Porridge, oatmeal, gruel, cereals, muesli, unsweetened
Diet sodas	Diet coke or diet sodas, flavored water included (no added sugar)
Milk	Skimmed, semi-skimmed and whole milk, with added sugar or not
Whole grain bread	Whole grain bread, dark roll, dark crispbread
Boiled or poached eggs	Boiled or poached eggs
Yogurt	Skimmed, semi-skimmed, or whole yogurt or kefir, with added sugar or not
Fruit with added sugar	Fresh fruit (also freshly squeezed, fruit smoothie) with added sugar
Cooked vegetables, potatoes	Cocked vegetables (e.g. cabbage, broccoli, green bean) and potato (also mixed)
Fresh meat (not fried)	Fresh meat (not fried) (chops, steak, bovine, pork, poultry, etc.)
Pasta, rice	Pasta, rice, noodles
Cheese	Cheese (cut, spreadable, grated)
Water	Tap and mineral water
Fruit juices	Fruit and vegetable juices (e.g. orange juice, apple juice)
Sweetened drinks	Sweetened drinks including sports drinks, bottled tea, syrup-based drinks
Legumes	Legumes (lentils, chickpeas, beans)
Fermented milk beverages	Skimmed, semi-skimmed or whole milk fermented drinks
Sweetened breakfast cereals	Sweetened or sugar added breakfast cereals and sweetened crisps, muesli
Butter, margarine on bread	Butter, margarine on bread
Fried or scrambled eggs	Fried or scrambled eggs
White bread	White bread, white roll, white crispbread
Mayonnaise	Mayonnaise and mayonnaise-based products
Sugary milk derivatives	Sugary milk derivatives such as milk cream, milk shakes
Fried fish	Fried fish and fish fingers
Fast food	Hamburgers, hot dogs, kebabs, wraps, falafel
Candies and trinkets	Snacks like candies, loose candies, marshmallows
Ice creams	Ice cream, milk or fruit-based bars
Sweetened starchy snacks	Snacks like biscuits, packaged cakes, pastries, puddings, cereal bars
Added sugar	Added sugar, honey, soluble cocoa, jam, quince
Chocolate, candy bars	Snacks like chocolate, candy bars
Pizza as main dish	Pizza as main dish

**Food item**	**Foods included**
Meat cold cuts	Cold cuts, preserved, ready to cook meat products
Ketchup	Ketchup (also as a topping on fries)
Fried potatoes	Fried potatoes, chips
Nuts, seeds, dried fruit	Nuts, seeds, dried fruit
Fried meat	Fried meat (chops, steak, bovine, pork, poultry, etc.)
Chocolate- or nut-based spreads	Chocolate or nut-based spreads
Savory pastries, fritters	Snacks like savory pastries and fritters
Olive and sunflower oils	Olive and sunflower oils
Salty snacks	Snacks like crisps, popcorn, etc.

Table S2. Relative food and beverages frequencies of consumption by dietary cluster in Spanish children and adolescents (GENOBOX study).

	**Health Conscious (*n*=403) *Z*-scores (mean (SD)**	**Sweet and Processed (*n*=271) *Z*-scores (mean (SD)**	p^a
Raw vegetables	0.23 (1.10)	-0.24 (0.81)	**<0.001**
Fruit with no added sugar	0.17 (1.05)	-0.27 (0.89)	**<0.001**
Hot drinks	0.15 (1.21)	-0.18 (0.63)	**<0.001**
Fish (not fried)	0.15 (1.02)	-0.12 (1.00)	**<0.001**
Unsweetened breakfast cereals	0.14 (1.16)	-0.16 (0.78)	**<0.001**
Diet sodas	0.13 (1.21)	-0.14 (0.65)	**<0.001**
Milk	0.13 (0.98)	-0.13 (1.06)	**0.001**
Whole grain bread	0.12 (1.06)	-0.15 (0.89)	**<0.001**
Boiled or poached eggs	0.12 (1.08)	-0.07 (0.95)	**0.014**
Yogurt	0.12 (1.14)	-0.13 (0.84)	**0.001**
Fruit with added sugar	0.10 (1.21)	-0.10 (0.73)	**0.006**
Cooked vegetables, potatoes	0.08 (1.06)	-0.10 (0.94)	**0.015**
Fresh meat (not fried)	0.02 (0.98)	-0.07 (1.04)	0.202
Pasta, rice	0.01 (1.05)	0.07 (1.00)	0.505
Cheese	0.01 (1.01)	0.07 (1.04)	0.446
Water	0.00 (0.99)	-0.02 (1.03)	0.772
Fruit juices	-0.03 (1.02)	0.18 (1.00)	**0.006**
Sweetened drinks	-0.03 (1.03)	0.11 (1.02)	0.066
Legumes	-0.04 (0.97)	0.12 (1.07)	**0.029**
Fermented milk beverages	-0.05 (1.00)	0.15 (1.03)	**0.008**
Sweetened breakfast cereals	-0.07 (0.78)	-0.05 (0.78)	0.819
Butter, margarine on bread	-0.08 (0.94)	0.21 (1.13)	**<0.001**
Fried or scrambled eggs	-0.09 (0.97)	0.16 (1.05)	**0.001**
White bread	-0.18 (0.88)	0.22 (1.10)	**<0.001**
Mayonnaise	-0.18 (0.73)	0.33 (1.27)	**<0.001**
Sugary milk derivatives	-0.19 (0.75)	0.3 (1.24)	**<0.001**
Fried fish	-0.19 (0.93)	0.28 (1.06)	**<0.001**
Fast food	-0.19 (0.69)	0.38 (1.29)	**<0.001**
Candies and sweets	-0.19 (0.83)	0.33 (1.17	**<0.001**
Ice creams	-0.19 (0.62)	0.17 (0.91)	**<0.001**
Sweetened starchy snacks	-0.21 (0.82)	0.33 (1.11)	**<0.001**
Added sugar	-0.22 (0.75)	0.36 (1.21)	**<0.001**
Chocolate, candy bars	-0.22 (0.77)	0.32 (1.18)	**<0.001**
Pizza as main dish	-0.23 (0.78)	0.37 (1.23)	**<0.001**
Meat cold cuts	-0.24 (0.81)	0.38 (1.17)	**<0.001**
Ketchup	-0.24 (0.58)	0.26 (0.85)	**<0.001**
Fried potatoes	-0.25 (0.89)	0.48 (1.04)	**<0.001**

	Health Conscious (n=403) Z-scores (mean (SD)	Sweet and Processed (n=271) Z-scores (mean (SD)	p^a
Raw vegetables	0.23 (1.10)	-0.24 (0.81)	**<0.001**
Fruit with no added sugar	0.17 (1.05)	-0.27 (0.89)	**<0.001**
Hot drinks	0.15 (1.21)	-0.18 (0.63)	**<0.001**
Fish (not fried)	0.15 (1.02)	-0.12 (1.00)	**<0.001**
Unsweetened breakfast cereals	0.14 (1.16)	-0.16 (0.78)	**<0.001**
Diet sodas	0.13 (1.21)	-0.14 (0.65)	**<0.001**
Milk	0.13 (0.98)	-0.13 (1.06)	**0.001**
Whole grain bread	0.12 (1.06)	-0.15 (0.89)	**<0.001**
Boiled or poached eggs	0.12 (1.08)	-0.07 (0.95)	**0.014**
Yogurt	0.12 (1.14)	-0.13 (0.84)	**0.001**
Fruit with added sugar	0.10 (1.21)	-0.10 (0.73)	**0.006**
Cooked vegetables, potatoes	0.08 (1.06)	-0.10 (0.94)	**0.015**
Fresh meat (not fried)	0.02 (0.98)	-0.07 (1.04)	0.202
Pasta, rice	0.01 (1.05)	0.07 (1.00)	0.505
Cheese	0.01 (1.01)	0.07 (1.04)	0.446
Water	0.00 (0.99)	-0.02 (1.03)	0.772
Fruit juices	-0.03 (1.02)	0.18 (1.00)	**0.006**
Nuts, seeds, dried fruit	-0.26 (0.76)	0.43 (1.17)	**<0.001**
Fried meat	-0.26 (0.83)	0.43 (1.10)	**<0.001**
Chocolate- or nut-based spreads	-0.27 (0.59)	0.46 (1.34)	**<0.001**
Savory pastries, fritters	-0.27 (0.68)	0.46 (1.28)	**<0.001**
Olive and sunflower oils	-0.31 (0.90)	0.39 (1.01)	**<0.001**
Salty snacks	-0.38 (0.58)	0.53 (1.12)	**<0.001**

[a] *p*: significance of the Student's t test, assessing differences between clusters. Bold letters in p values mean significant differences between dietary clusters. Abbreviations: SD, standard deviation.

Table S3. Main differences related to cardiometabolic risk indicators and health between dietary clusters in age and gender subgroups of children and adolescents (GENOBOX study).

	**Age group**				**Gender**			
	**Children (*n*=439)**		**Adolescents (*n*=235)**		**Male (*n*=307)**		**Female (*n*=367)**	
	**Health Conscious (*n*=245)**	**Sweet and Processed (*n*=194)**	**Health Conscious (*n*=158)**	**Sweet and Processed (*n*=77)**	**Health Conscious (*n*=175)**	**Sweet and Processed (*n*=132)**	**Health Conscious (*n*=228)**	**Sweet and Processed (*n*=139)**
Age (years)	9.2 (1.7)	9.1 (1.8)	13.5 (1.0)	13.2 (1.0)	10.8 (2.5)	10.5 (2.5)	**11.0 (2.5)**	**10.1 (2.3)*****
Body composition indicators								
BMI (kg/m^2)	**23.2 (4.4)**	**21.7 (5.4)****	26.8 (5.2)	26.9 (6.7)	**24.4 (4.9)**	**23.0 (6.7)***	24.7 (5.1)	23.3 (5.8)
BMI Z-score (kg/m^2)	**1.95 (1.59)**	**1.47 (1.86)***	1.99 (1.49)	2.06 (1.91)	**2.15 (1.72)**	**1.67 (2.13)****	1.83 (1.39)	1.62 (1.63)
Body mass (kg)	**45.3 (13.0)**	**41.7 (14.8)***	69.1(16.3)	69.0 (20.2)	53.9 (18.6)	50.5 (22.7)	55.2 (18.4)	486 (18.4)
Hip circ. (cm)	**83.9 (10.9)**	**78.7 (12.4)****	99.7 (11.7)	95.9 (15.4)	**88.2 (12.7)**	**82.1 (15.8)****	91.7 (14.0)	84.2 (14.5)
Waist circ. (cm)	**76.9 (12.3)**	**72.4 (14.6)***	90.6(14.9)	89.7(18.9)	82.5 (14.9	77.5 (18.5)	82.2 (15.0)	76.8 (16.8)
Waist to hip index	0.91 (0.07)	0.90 (0.08)	0.90 (0.09)	0.90 (0.10)	0.93 (0.07)	0.91 (0.08)	**0.89 (0.10)**	**0.88 (0.10)***
Waist to height index	0.56 (0.08)	0.53 (0.09)	**0.57 (0.09)**	**0.56 (0.11)***	0.57 (0.08)	0.54 (0.10)*	0.56 (0.10)	0.54 (0.10)
Skinfold sum (mm)	**81.8 (30.9)**	**63.8 (33.6)*****	87.1 (28.3)	81.9 (39.0)	**81.4 (33.8)**	**64.4 (38.0)****	85.3 (27.0)	71.7 (33.0)
FMI Z-score (kg/m^2)	**6.4 (3.3)**	**4.9 (3.9)***	8.3 (5.0)	7.8 (4.6)	8.7 (4.9)	6.8 (5.2)	6.1 (3.1)	4.6 (2.8)
FFMI Z-score (kg/m^2)	10.2 (4.9)	9.2 (5.3)	13.7 (4.8)	13.8 (4.9)	12.0 (5.2)	10.7 (6.3)	11.4 (5.1)	10.0 (4.6)
Cardiometabolic indicators								
Blood pressure								
SBP (mm Hg) ^	106 (12)	105 (13)	113 (14)	115 (14)	108 (13)	107 (13)	110 (14)	108 (15)
DBP (mm Hg) ^	64 (11)	65 (10)	**65 (10)**	**68 (10)***	**65 (10)**	**66 (10)***	65 (11)	66 (10)
General metabolic biomarkers								
Glucose (mg/dL)	83 (8)	85 (8)	85 (8)	87 (7)	85 (8)	86 (8)	84 (8)	85 (7)
Insulin (mU/L)	10.53 (6.90)	9.63 (9.34)	14.78 (9.86)	16.23 (9.81)	11.27 (9.14)	10.46 (10.92)	12.89 (7.80)	12.53 (8.78)

	Age group				Gender			
	Children (n=439)		Adolescents (n=235)		Male (n=307)		Female (n=367)	
	Health Conscious (n=245)	Sweet and Processed (n=194)	Health Conscious (n=158)	Sweet and Processed (n=77)	Health Conscious (n=175)	Sweet and Processed (n=132)	Health Conscious (n=228)	Sweet and Processed (n=139)
HOMA-IR	2.2 (1.50)	2.1 (2.0)	3.1 (2.2)	3.5 (2.2)	2.40 (2.00)	2.28 (2.39)	2.70 (1.70)	2.68 (1.94)
TG (mg/dL)	67 (34)	66 (33)	73 (33)	77 (36)	67 (37)	63 (34)	71(31)	74 (35)
Cholesterol (mg/dL)	168 (31)	163 (28)	161 (28)	155.6 (25.7)	164 (30)	164 (29)	**166 (30)**	**159 (26)***
LDLc (mg/dL)	98 (27)	92 (24.8)	96 (25)	89 (24)	97 (26)	93 (27)	**98 (27)**	**90 (23)***
HDLc (mg/dL) ^	52 (13)	57 (16)	46 (12)	49 (12)	50 (13)	57 (16)	49 (13)	53 (15)
HDLc/LDLc index	0.67 (0.56)	0.85 (0.70)	**0.52 (0.22)**	**0.73 (0.61)***	0.67 (0.6)	0.8 (0.6)	**0.57 (0.32)**	**0.82 (0.74)***
AST (U/L)	24 (9.8)	24 (6)	19 (7)	22 (10)	23 (8)	24 (7)	20 (9)	23 (7)
ALT (U/L)	**21 (13)**	**18 (6.1)****	19 (11)	21 (19)	**21 (12)**	**18 (8)***	19 (11)	19 (14)
GGT (U/L)	**12 (5)**	**12 (6.2)***	12 (9)	15 (11)	13 (9)	13(5)	11 (5)	13 (9)
Oxidative stress biomarkers								
Carotenes / TG	**2.01 (1.09)**	**1.62 (1.25)***	1.28 (1.16)	1.26 (1.23)	1.99 (2.06)	1.77 (1.38)	1.51 (1.27)	1.25 (1.05)
Tocopherols / TG	0.15 (0.07)	0.15 (0.07)	0.12 (0.07)	1.38 (0.06)	0.15 (0.07)	0.16 (0.07)	0.14 (0.07)	0.14 (0.06)
TAC (mM Eq Trolox®)	1.82 (0.79)	2.02 (0.86)	2.37 (0.90)	2.28 (1.00)	2.00 (0.86)	2.05 (0.87)	2.08 (0.89)	2.13 (0.95)
Catalase (U/g Hb)	**155.21 (77.63)**	**164.64 (174.66)****	181.65 (137.56)	161.91 (61.95)	158.50 (87.03)	169.62 (115.34)	160.89 (114.11)	166.70 (182.98)
Adipokines and biomarkers of inflammation and endothelial damage								
Adiponectin (mg/L)	15.41 (9.1)	15.29 (7.92)	**12.91 (7.44)**	**14.89 (9.50)***	14.39 (9.67)	15.11 (7.35)	14.73 (7.80)	15.24 (9.15)
Leptin (ug/L)	**13.1 (9.8)**	**12.94 (18.85)*****	19.56 (16.00)	17.79 (15.37)	**13.8 (12.18)**	**13.45 (16.99)*****	16.96 (13.42)	14.68 (14.72)
Resistin (ug/L) ^	20.03 (15.53)	20.89 (14.12)	20.60 (13.92)	21.91 (15.48)	19.35 (15.87)	21.00 (16.01)	20.86 (14.06)	21.13 (12.91)
TNFα (ng/L) ^	2.99 (1.75)	2.93 (1.63)	**2.48 (1.58)**	**2.70 (1.26)***	**3.02 (1.55)**	**3.15 (1.7)***	2.66 (1.84)	2.64 (1.41)
MCP-1 (ng/L)	**88.70 (38.16)**	**93.99 (41.75)***	**87.75 (36.98)**	**88.22 (27.66)****	**89.73 (31.5)**	**98.29 (38.15)****	87.45 (42.08)	87.63 (38.94)
tPAI-1 (ug/L)	**22.29 (14.28)**	**23.95 (16.89)***	23.93 (14.16)	30.34 (17.81)	**20.81 (12.78)**	**24.84 (17.48)*****	24.6 (15.23)	26.09 (17.20)

	**Age group**				**Gender**			
	**Children (*n*=439)**		**Adolescents (*n*=235)**		**Male (*n*=307)**		**Female (*n*=367)**	
	**Health Conscious (*n*=245)**	**Sweet and Processed (*n*=194)**	**Health Conscious (*n*=158)**	**Sweet and Processed (*n*=77)**	**Health Conscious (*n*=175)**	**Sweet and Processed (*n*=132)**	**Health Conscious (*n*=228)**	**Sweet and Processed (*n*=139)**
Selectin (ug/L) ^	29.40 (15.00)	32.36 (17.22)	25.00 (14.10)	24.33 (13.07)	30.41 (13.9)	28.35 (14.4)	24.81 (14.97)	32.46 (18.51)
sVCAM1 (mg/L) ^	**1.07 (0.28)**	**1.19 (0.25)****	1.01 (0.35)	0.98 (0.19)	1.05 (0.36)	1.13 (0.27)	**1.02 (0.28)**	**1.14 (0.24)***
MPO (ug/L)	43.63 (96.63)	35.30 (44.47)	34.50 (57.02)	26.06 (34.74)	45.36 (116.55)	37.39 (49.61)	**35.62 (41.37)**	**28.75 (33.64)***

p: significance of the test used to assess differences between dietary clusters. *: p≤0.05; **: p≤0.01; ***: p≤0.001. Bold letters in p values mean significant differences between dietary clusters. ^ Logarithm transformed variable used for analyses T-Student's test was used to analyze differences related to age. Stepwise generalized linear models were adjusted for recruitment center, sport practice, pubertal stage, gender, and, additionally, maternal education level for body composition or BMI Z-score for metabolic variables. Abbreviations: BMI: body mass index; circ.: circumference; FMI: fat mass index; FFMI, free fat mass index: SBP: systolic blood pressure; DBP: diastolic blood pressure; HOMA-IR: homeostatic model assessment for insulin resistance; TAG: triglycerides, HDLc: high-density lipoprotein cholesterol; LDLc: low-density lipoprotein cholesterol; AST: aspartate transaminase; ALT: alanine transaminase; GGT: gamma-glutamyl transferase; TAC: total antioxidant capacity; TNFα: tumor necrosis factor alpha; MCP-1: monocyte chemoattractant protein-1; tPAI-1: total plasminogen activator inhibitor-1; sVCAM1: soluble vascular cell adhesion molecule-1; MPO: myeloperoxidase.
